# Severe polymyositis occurring in a cancer patient directly after chemotherapy: etiology and management

**DOI:** 10.2144/fsoa-2021-0011

**Published:** 2021-06-30

**Authors:** Charles Soutif, Thaïs Tison, Isabelle Focant, Emmanuel Seront

**Affiliations:** 1 Department of medical oncology, Hopital de Jolimont, Haine Saint Paul, 7100, Belgium; 2 Department of rheumatology, Hopital de Jolimont, Haine Saint Paul, 7100, Belgium

**Keywords:** colorectal cancer, high-dose steroids, immunoglobulins, polymyositis

## Abstract

A 72-year-old woman was diagnosed with metastatic colorectal cancer and treated with oxaliplatin-based chemotherapy and bevacizumab. One week after the second administration of chemotherapy, she presented acute-onset dysphagia and rapidly progressing proximal muscle weakness, associated with elevation of the creatinine phosphokinase enzymes. Magnetic resonance imaging raised suspicion of polymyositis. Etiology remained unclear but paraneoplastic origin or immune modulation by chemotherapy was considered. High-dose methylprednisolone and intravenous immunoglobulins were started with continuation of chemotherapy. Although there was rapid normalization of muscle enzyme, the general status deteriorated rapidly with aggravation of dysphagia, complete immobilization and death. This case highlights the importance of considering muscle weakness as paraneoplastic syndrome or drug-induced toxicity in colorectal cancer patients. Despite aggressive management, prognosis remains poor.

## Lay abstract

During cancer treatment, nonspecific symptoms can occur, such as deglutition trouble and muscle weakness. These symptoms could be related to treatment (drug induced-toxicity) or to cancer (paraneoplastic syndrome). Early recognition of these symptoms is important as they can rapidly increase and impact negatively the outcome of patients. We report the case of a myositis with unclear etiology. Despite aggressive management with intravenous immunoglobulins, the patient died very rapidly.

Colorectal cancer is the third most common malignancy worldwide with an incidence of approximately 1.4 million new cases and almost 700,000 deaths in 2012 [1]. The early stage can be treated with curative intent. The advanced stage remains a challenge for clinicians and is associated with poor outcome; however, major improvements have been made with the development of new strategies and tailored genomic-based treatments. These anticancer agents are associated with cumulative toxicities and can deteriorate life quality. In addition to direct effects of cancer on patient status, indirect manifestations can occur, as observed in paraneoplastic syndromes, which are not related to tumor mass, but rather related to the production of functional peptides/hormones and cross reactivity that can occur between tumor and host antigens. Paraneoplastic syndromes can affect most of the organs and the symptoms are sometimes not specific and difficult to distinguish from cancer-related symptoms or treatment-induced toxicities. Polymyositis are autoimmune disorders characterized by inflammatory myopathy belonging to the group of connective tissue disorders. Even if frequently idiopathic, they are often associated with malignancy; manifestations can occur before or after cancer diagnosis, rendering the diagnosis challenging in some cases [2]. We report the case of a patient with metastatic colorectal cancer who presented asthenia, muscle weakness and deglutition trouble early after chemotherapy initiation. The suspicion of paraneoplastic syndrome rather than drug-induced toxicity led us to continue chemotherapy and rapidly start steroids and intravenous immunoglobulins.

## Case presentation

**Figure 1.**
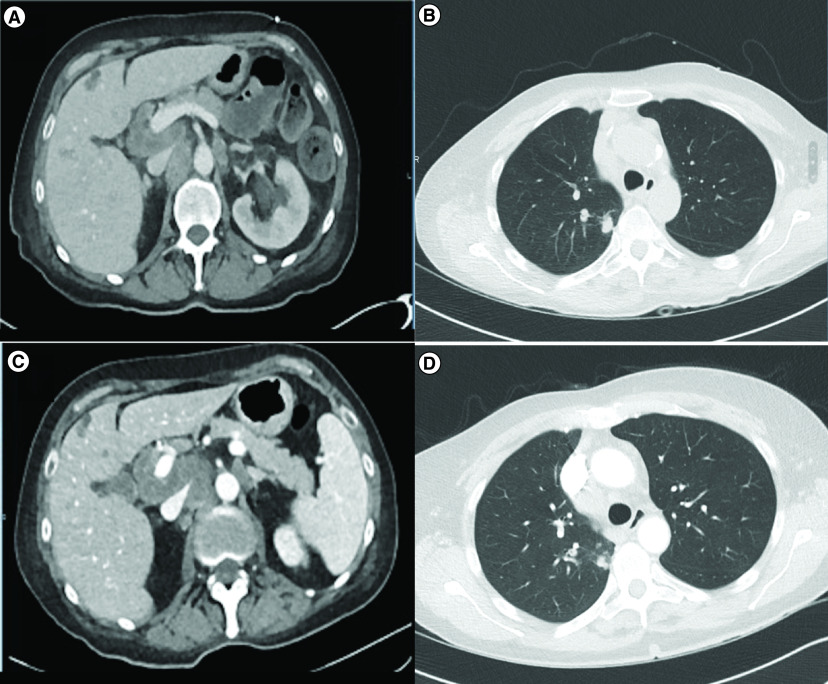
Radiological evolution during treatment course. **(A & B)** Abdominal and thoracic imaging at diagnosis showing abdominal lymph nodes, liver metastasis and lung metastases. **(C & D)** Abdominal and thoracic imaging performed 3 months after chemotherapy onset and concomittant to myositis symptomatology, showing stable disease based on RECIST criteria.

In June 2020, a 72-year-old woman presented with acute abdominal pain. Thoraco-abdominal computed tomography showed a bowel occlusion and a right colic tumor associated with liver and lung lesions (Figure 1A & B). Resection of the tumor relieved occlusion and histological findings confirmed moderately differentiated K-Ras-mutated colic adenocarcinoma. Her past medical history included a nonerosive rheumatoid arthritis diagnosed in July 2009, with positive anti-CCP antibodies (80 U/ml), clinically well controlled on weekly ledertrexate (15 mg weekly) treatment in association with folic acid. There was no history of smoking or alcohol intake. Ledetrexate was stopped 5 days before starting the first cure of chemotherapy and there was no other medication history.

In August 2020, oxaliplatin-based chemotherapy was initiated in association with bevacizumab (on a 2-week scheme) and the first administration was well tolerated without any adverse event except grade 2 asthenia that was related to chemotherapy. Seven days after the second administration, she reported acute-onset deglutition troubles and proximal muscle weakness, associated with mild diffuse myalgia. These symptoms worsened rapidly in one week, resulting in incapacity to stand up, walk, raise upper and lower limbs and swallow saliva. At presentation in emergency, the patient was completely immobilized in her bed. There was no joint pain, no fever, no vision trouble and no respiratory difficulty.

Cardiopulmonary auscultation was normal, as was the cutaneous examination. Neurological examination showed grade 4 Medical Research Council bilateral proximal limb weakness. There was no ptosis, no diplopia and no oculomotricity defect. There was no sign of fatigability and weakness did not improve at rest. Deep tendon reflexes were normal and symmetric, as was sensitive testing.

Laboratory assays revealed normal blood counts, normal renal, liver and thyroid function, and absence of vitamin deficiency. C-reactive protein (CRP) was elevated to 40 mg/dl (normal range: 0–15 mg/dl). Creatinine phosphokinase (CPK) was elevated to 8892 IU/l (normal range: 26–192). Serologies for Ebstein–Barr virus, cytomegalovirus, HIV, hepatitis B, hepatitis C and Lyme disease were negative. The antinuclear antibody was titrated at 1/160 without any specificity (U1 NRP, scl-70, SS-A, SS-B, CENP-B, Sm, JO-1), Ac anti-DNA, extractable core antigens, antineutrophil cytoplasmic antibodies, antiphospholipid syndrome antibodies and ANCA, were all negative. Anti-CCP was elevated at 69 U/ml. Blood electrophoresis and urinary analysis were normal.

The patient underwent cerebral MRI that was normal; there were no cerebellar or trunk lesion compatible with stroke or metastases. The thoraco-abdominal computed tomography showed mild regression of liver and lung tumor lesions (-10% following RECIST v1.1 criteria, Figure 1 C & D). The electrocardiogram was normal and cardiac ultrasonography did not show any ventricular dysfunction. CPK continued to rise during the next 3 days despite hydration, reaching 10,000 IU/l; renal function remained within normal range.

**Figure 2.**
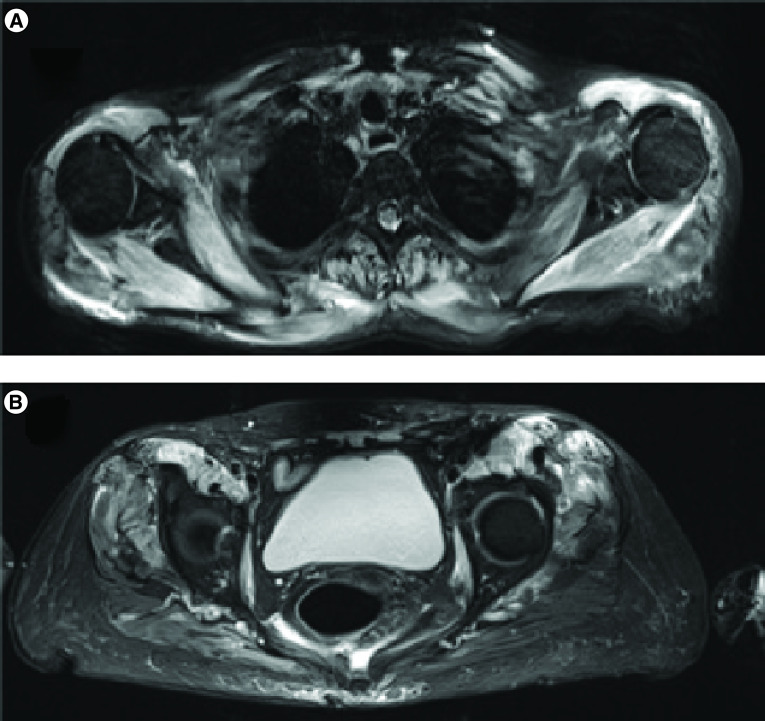
Magnetic resonance imaging showing myositis. Hyperfixation of proximal muscles confirms inflammatory proximal myopathy. **(A)** Upper limb. **(B)** Lower limb.

A T1, T2 Fat Sat MRI of the thighs, shoulders and proximal limb showed muscle hyperfixation (Figure 2). The spirometry demonstrated a restrictive pulmonary syndrome with a total lung capacity corresponding to 60% of the expected value. The electromyography (EMG) was normal but was performed on peripheral and distal muscle, and did not explore proximal muscle. Repetitive stimulation did not show any decrement of the muscle action potential. Due to the poor degrading general condition of the patient, and as MRI findings were strongly suggestive of an inflammatory myositis, a muscle biopsy was not proposed to the patient. The important elevation of CPK and the muscle fixation on MRI strongly supported a polymyositis diagnosis. The immune history of our patient, the absence of improvement despite the prolonged chemotherapy interval-free and the close time relationship with cancer diagnosis led us to suspect a paraneoplastic syndrome rather than drug-induced toxicity.

The absence of falls, hematoma and renal failure, as well as the absence of CPK decrease on hydration excluded rhabdomyolysis. Neuromuscular transmission anomalies have been considered but the elevation of CPK, the absence of muscle weakness that progressively increases with repetitive muscle action but decreases with rest, absence of specific auto-antibody and the nonpathognomonic EMG was not in favor of Myasthenia gravis or Lambert–Eaton. Other causes of myositis such as electrolyte imbalance (hypokaliemia, hypomagnesemia or hypophosphatemia) or hormonal disturbance (hypercorticism, hypothyroidism or diabetes) were excluded; there was no chronic use of steroids, statins, azathioprine.

Due to the difficulties with swallowing saliva and evacuating the bronchio-alveolar secretion, we started glycopyrronium intravenously (IV) at the dose of 1.2 mg daily, which rapidly improved the amount of secretions. Intravenous methylprednisolone high doses (1000 mg daily for 3 days) was initiated with, 4 days later, decrease in CPK levels (3000 IU/l) but no clinical benefit in term of muscle weakness and deglutition trouble was observed; chemotherapy (oxaliplatin-based therapy plus bevacizumab) was thus restarted.

Seven days after the methylprednisone initiation, CPK were normalized and did not increase again after chemotherapy administration; however, due to the absence of weakness improvement and aggravation of deglutition trouble, intravenous immunoglobulins (IVIGs) (1 g/kg daily for 2 days) was administered. The general status of our patient deteriorated, with it proving impossible to swallow, alongside the accumulation of pulmonary secretions and impossibility for the patient to move any part of her body. Our patient died 4 weeks after the start of methylprednisone and 6 weeks after the initial manifestations.

## Discussion

The diagnosis of polymyositis in this case has been made based on the highly suggestive clinical features, the high elevation of CK without other explanations, and the muscle MRI. EMG was considered as normal; however, EMG was done early after apparition of symptoms and it explored distal limb part, which could render this result not clearly significant [3]. Biopsy was not performed in our patient due to the poor general status and the poor indicator value at this stage. New insight in inflammatory myositis have defined new criteria for idiopathic inflammatory myopathy. Those criteria are based on 12 clinical parameters transformed into a numerical value: age; sex; pattern of weakness; signs of skin manifestations; laboratory features; and four facultative biopsy parameters [4]. The score was up to 6.5–8.4 in this case, and classified the disease as ‘definite inflammatory myopathy’, even in absence of biopsy and significant EMG.

The etiology of this myopathy remains unclear in this patient but different hypotheses can be advanced such as drug-induced myopathy, paraneoplastic syndrome and immune-modulation by chemotherapy.

The rapid occurrence of symptoms after the first chemotherapy administration may invite suspicion of drug toxicity. Even if imbalance between antiangiogenic and proangiogenic VEGF isoforms in inflammatory myopathies has been described, the role of the anti-VEGF bevacizumab in myositis development remains unknown and no case has been described; one case of myositis has been described with sorafenib [5,6]. Furthermore, the absence of symptomatology improvement with the chemotherapy interval-free and the absence of CPK increase after the second administration of chemotherapy seem not consistent with this etiology.

The association between cancer and inflammatory myopathy, such as polymyositis or dermatomyositis, has been widely reported but the pathogenesis remains elusive [2]. It was reported that cancer can occur between the 2 years preceding and the 3 years following the diagnosis of polymyositis. The close time relation of clinical manifestations with cancer diagnosis led us to suspect paraneoplastic syndrome, even if polymyositis is rarely described in colorectal cancer. Gkegkes *et al.* reviewed the profile of dermato-polymyositis in 27 patients with colorectal cancer and found that in 80% of patients, symptoms manifest before the diagnosis of cancer [7]. Pathophysiology of paraneoplastic polymyositis remains unknown but immune dysfunction seems to play a key role in the development of paraneoplastic syndrome. An immunological response is elicited by the ectopic expression of intra- or extracellular antigens by the tumor, which leads to cross-reactivity between the tumor cells and components of the neuromuscular system. This creates antitumoral antibodies that attack the muscle.

The short interval between chemotherapy and symptoms occurrence could also be explained by the concept of immune modulation by chemotherapy. Chemotherapy, by killing cancer cells, reloads multiple neoantigen in the circulation and stimulates the T-cell activation. In addition, chemotherapy, by its cytotoxic activity, depletes the immune suppressive cells such as T-regulatory cells. Chemotherapy could thus have triggered this immune-related syndrome in our patient with known history of immune-related polyarthritis [8].

Another review demonstrated that specific autoantibodies are detected in around 40% of patients with paraneoplastic syndromes [9,10]. TIF1-γ is a myositis-specific autoantibody, as it was not detected in other autoimmune diseases or in other noninflammatory myopathies. It could really help to differentiate a cancer-associated myositis from another etiology. Its negative and positive predictive value of presence for a diagnosis of cancer-associated myositis reaches 92 and 66.7%, respectively. This suggests that, in the diagnosis of a myositis, negative TIF1-γ reasonably rules out the presence of associated cancer. Unfortunately, this serologic testing was not assessed in our case [11]. Antinuclear antibody was considered as negative in our patient, which may be consistent with a retrospective cohort study of patients with polymyositis that demonstrated a strong association between antinuclear antibody positivity and lower likelihood of malignancy [12].

When considering paraneoplastic or cancer-associated etiology, polymyositis is a medical emergency and early recognition can allow early and aggressive management. Even if cancer treatment often leads to symptom improvement in paraneoplastic polymyositis [10], other strategies have to be considered and include high dose steroids, immunosuppressive agents (IVIGs).

Despite the lack of evidence from randomized trials, corticosteroids remain the agents of choice for inflammatory myopathy. Considering the severity of the disease, high doses of steroid were started as the first-line treatment in our patient. This resulted in CPK decrease but no clinical benefit. Rapid response to corticosteroid therapy has been associated with a favorable prognosis [13,14]. The introduction of immunosuppressive agents is usually considered in case of poor response to corticotherapy or rapidly progressive disease. The most frequent immunosuppressive agents include methotrexate, azathioprine or cyclosporine [15]. In our case, we decided to continue chemotherapy in order to control the primary cause and to induce immunosuppression. It is interesting to note that our patient was previously treated with ledetrexate for rheumatoid arthritis. Ledertrexate was interrupted just before starting chemotherapy. Whether ledertrexate could have hid polymyositis symptoms before cancer diagnosis remains unknown. The absence of benefit led us to consider IVIG, that resulted in efficacy in refractory cases of polymyositis despite lack of large clinical trials. IVIGs was shown to improve the muscle strength in up to 70% of patients with inflammatory myositis, particularly swallowing muscles and this benefit is more important in patients with early disease [16–18]. Marie *et al.* reported that IVIG, when used in first-line setting, could improve symptoms in 88% of polymyositis patients with life-threatening esophageal manifestations [19]. IVIGs in our patient were unsuccessful; whether earlier administration of IVIG (in the same time of high doses of steroids) could have improved prognosis of our patient remains unknown.

## Conclusion

In conclusion, recognition of paraneoplastic myositis remains difficult but should also be considered, even in case of cancer stability on anticancer treatment. Early recognition could facilitate a more successful treatment outcome and immediate use of steroids in association of IVIGs should be considered.

## Future perspective

Cancer-associated polymyositis is a challenge for clinicians. Many improvements need to be done in detecting specific auto-antibodies to identify the etiology of myositis. The better understanding of the immune-related pathways implicated in this pathology will help to develop new agents specifically targeting this entity, even if the main treatment remains the cancer management. We hope that the improvement in therapeutic strategies will lead to a better control of the cancer and in the subsequent cancer-associated syndrome. Even though MRI can help to identify the polymyositis, future imaging modalities will also help to better clarify the involvement of polymyositis.

**Executive summary**

- Muscle weakness associated with elevation of creatinine phosphokinase should raise suspicion of myositis. Dysphagia can reflect a grievous sign. Paraneoplastic syndrome such as polymyositis or dermatopolymyoisitis should always be suspected. Anti-TF1 gamma can help to rule out cancer.
- Magnetic imaging resonance can lead to rapid diagnosis and should be part of the diagnostic modalities.
- Patients with polymyositis, particularly in paraneoplastic syndrome, have to be closely followed as clinical degradation can rapidly progress till death.
- High-dose methylprednisolone and intravenous immunoglobulins should be started rapidly in polymyositis with life-threatening complications.
